# The ‘GROW Social Network’ datasets

**DOI:** 10.21307/connections-2019.017

**Published:** 2020-02-13

**Authors:** Sabina B. Gesell, Evan C. Sommer, Shari l. Barkin

**Affiliations:** 1 Department of Social Sciences and Health Policy, Department of Implementation Science, Wake Forest School of Medicine, Winston-Salem, North Carolina.; 2 Department of Pediatrics, Vanderbilt University Medical Center, Nashville, Tennessee.

**Keywords:** Social networks, Hispanic, Healthy lifestyle, Randomized controlled trial

## Abstract

The GROW Social Network datasets were compiled as part of a 3-year community-based family-based pediatric obesity prevention intervention (N = 610). The datasets include (i) multiplex edges between adult study participants at four timepoints (baseline, 3, 12, and 36 mon), and (ii) multiplex edges within small intervention-only subgroups (30 groups of approximately 10 adult intervention participants) and a previously validated self-report measure of perceived cohesion at three timepoints (3, 6, and 12 wk). Actor attributes are richly characterized in a linkable dataset.

## Overview

The Growing Right Onto Wellness (GROW) social network datasets were collected as part of the longest pediatric obesity prevention intervention for low-income families. GROW was a 3-year randomized controlled trial in Nashville, Tennessee, USA conducted in 2014 to 2017 in conjunction with that city’s Department of Parks and Recreation (Barkin et al., 2018). Participants were 610 low-income, parent–child dyads, 90% of whom were Hispanic. Parent–child dyads were randomized to either (i) a behavioral intervention that used group educational sessions and motivational interviewing to build skills for healthy lifestyles (GROW Healthier), or (ii) a school readiness comparator group (GROW Smarter). However, both groups received the school readiness material so that the only difference between conditions was the obesity prevention intervention. The intervention group promoted healthy lifestyle behaviors through three intervention phases: (i) the intensive phase delivered in a group format where small groups of the same participants met weekly for 12 90-min sessions over three months (3 mon timepoint); (ii) the maintenance phase, which included monthly phone-call coaching over nine months (12 mon timepoint); and (iii) the sustainability phase, which included cues to action to use the surrounding built environment for health over 24 months (36 mon timepoint).

Integrated within the GROW Healthier intervention was the intentional building of new social networks among intervention group participants during the intensive phase (weeks 1–12). During the intensive phase, social network diagnostics were used to guide intervention implementation and to intentionally create peer-to-peer interaction to spread new behaviors within small groups of parents (Valente, 2012).

A network survey identified each study participant’s advice network [‘In your GROW group, who would you go to outside of sessions for advice on making your family healthier (like being more active, eating healthier, and getting more sleep)?’]. Only intervention participants were asked this question. Relationships outside of sessions were measured to capture stronger personal ties, rather than the weaker associations created in a classroom setting, where all participants were structurally required to interact with each other. Responses were discussed with interventionists.

Multiple network diagnostics (isolates, density, centrality, subgroups, transitivity, cohesion) were computed to understand the social network features within each small intervention group by the midpoint of the intensive phase (week 6 of the 12-week intensive group phase). Using pre-determined thresholds defined in the Social Network Diagnostic Tool (Gesell et al., 2013), action reports were created with concrete recommendations for the interventionists to use in subsequent sessions. If a network was not forming at week 6 or if a network had structural signatures that did not align with the goal of group cohesion, the interventionist would receive a visualization of the network and concrete data-driven recommendations on how to augment group connectivity. Recommendations included: ‘Connect Participant 1 with any of these four group members’; ‘Make sure Participants 2 and 3 do not form a separate subgroup. Invite others to work with them in small group activities in session’; ‘Participants 4, 5, 6 have the strongest/most ties to other group members. In small group activities, pair them with Participants 7, 8, 9’; ‘Please call on Participants 1, 2, 3 to answer questions in session with the goal of not letting them fade into the background. Later in the session, refer back to what they said to show their input is valued’; ‘Participants 8 and 9 are not connected to anyone in their group. Please touch base with them in private to make sure they do not feel excluded. Please do not put them on the spot publically.’). This action report was then discussed with the interventionists, who were instructed to use the recommendations during the remaining sessions (weeks 7–12) to increase group cohesion. If the network was cohesive at week 6, as defined by the pre-defined thresholds per pre-determined network indicators above, then the interventionist would be instructed not to alter their teaching methods. Fidelity was assessed in more than 10% of all sessions and was high, at 99% adherence to protocol. Our Social Networks Diagnostic Tool for monitoring group dynamics is published in detail (Gesell et al., 2013). Intervention and control group members were always kept separate from each other to avoid contamination but participants all lived in the same city and connections could have existed or formed over time between individuals in the two groups despite efforts to avoid contamination. There was no network building component for the GROW Smarter comparator group.

The GROW study methods (Poe et al., 2013) and primary paper describing the intervention’s effect on child outcomes (Barkin et al., 2018) are published in detail. The study is registered at ClinicalTrials.gov (NCT01316653).

## Data collection

Data collection occurred between June 2014 and July 2017. Participants were ≥ 18 years, with a child 3 to 5 years who was not yet obese (BMI percentile ≥ 50 and < 95), English or Spanish speaking, qualified for a food assistance program, and living or frequently traveling within five miles of a participating recreation center. The Vanderbilt University Medical Center Institutional Review Board (IRB No. 120643) and an NHLBI-appointed Data and Safety Monitoring Board approved the study protocol and routinely evaluated both participant safety and protocol adherence.

Two network datasets are available: the Full Network Dataset and the Discussion and Advice Network Dataset. Both can be linked to a rich dataset of mediator, moderator, and outcome variables collected in the trial.

## Full network dataset

Participants: all adult study participants (*N*=610).

Data collection: data collection occurred at local community centers or at participants’ homes (as participants preferred) in the participants’ language of choice (English, Spanish). Data collectors read the questions out loud and captured participants’ responses in REDCap (Harris et al., 2009).

Social network measures: to identify social ties among adult study participants over the duration of the study, all participants responded to the following name generator question at each assessment:

1. ‘Please provide the names of up to 7 people you know and talk to from GROW (this can include anyone in GROW Smarter or GROW Healthier).’
  No roster was provided to participants, and there were no other restrictions on who they could nominate. For each nomination, participants were then asked this series of questions:
2. Did you know this person before starting the study? (0 – No, 1 – Yes).
3. How close do you feel to this person? (1 – Not close at all, 2 – Somewhat close, 3 – Very close).
4. Do you talk to this person (including in person, phone, e-mail, Facebook, text) about eating healthy outside of GROW activities? (0 – No, 1 – Yes).
5. Do you talk to this person (including in person, phone, e-mail, Facebook text) about physical activity outside of GROW activities? (0 – No, 1 – Yes).
6. Do you talk to this person (including in person, phone, e-mail, Facebook text) about sleep outside of GROW activities? (0 – No, 1 – Yes).
7. Do you talk to this person (including in person, phone, e-mail, Facebook text) about parenting outside of GROW activities? (0 – No, 1 – Yes).
8. Do you talk to this person (including in person, phone, e-mail, Facebook text) about media use (e.g. TV, computer) outside of GROW activities? (0 – No, 1 – Yes).
9. How often do you talk to this person? (1 – Never, 2 – Rarely, 3 – Sometimes, 4 – All the time).
10. Has this person helped you make changes to your lifestyle? (0 – No, 1 – Yes).

## Discussion and advice network dataset

Participants: Adult GROW Healthier intervention participants only (*N*=304).

Data collection: surveys were administered at the community recreation center to the intervention group participants in attendance. Data were collected at the beginning of the group sessions (intensive phase) in week 3, at the midpoint in week 6, and at the end in week 12. Study participants who were not in attendance on data collection days were allowed a second opportunity to complete the surveys in person at the subsequent sessions (weeks 4 and 7).

Social network measures: to ease respondent burden and to reduce measurement error, participants were provided with photos and names of other subgroup members. The intervention assistant read the name generator questions out loud. Participants placed stickers on the photo sheet to indicate their ties to other group members. This aided recall was necessary to reduce measurement error resulting from low literacy, partial names, and similar or identical names. This process helped distinguish individuals with the same or very similar names. A second trained study person reviewed all survey data to ensure their quality and accuracy. The name generator questions were:

1. ‘ In your GROW group, who would you go to outside of sessions for advice on making your family healthier (being more active, eating healthier, and getting more sleep)?’
2. ‘In your GROW group, with whom do you discuss these issues (being more active, eating healthier, and getting more sleep) outside of sessions?’

A six-item previously validated measure of perceived cohesion, reflecting two underlying dimensions of cohesion (sense of belonging, feelings of morale), was also administered (Bollen and Hoyle, 1990; Gesell et al., 2016). The intervention assistant read the items out loud, and respondents followed along and circled their responses.

These social network datasets can be linked to demographic and health behavior data stored in separate datasets including:

Adult descriptors collected at baseline: demographics (sex, age, race/ethnicity, age, education, income, acculturation, etc.), food security, perinatal health, family health history, genotype.

Adult and child health-related data were collected at baseline, 3, 9, 12, 24, and 36 mon and included height, weight, BMI, waist circumference, triceps skinfold, accelerometry, eating behaviors, sleep, media use, parenting practices, use of recreation center, use of library, perception of the built environment, stress, depression, goal setting and monitoring, executive functioning, weight perception, self-efficacy, readiness to change, child asthma/allergies, well-being, smoking, child healthcare. However, not all data were collected at all time points. See BioLINCC and published protocols for full details (Poe et al., 2013).

Importantly, social network and health behavior data were collected simultaneously at several timepoints: baseline, 3, 12, and 36 mon.

**Table:** 

	T1 baseline	Week 3 Intervention only	Week 6 Intervention only	T2 3 mon	T3 9 mon	T4 1 yr	T5 2 yr	T6 3 yr
Full network dataset	**X**			X		**X**		**X**
Discussion and advice network dataset		X^a^	X^a^	X^a^				
Adult and child health behavior dataset	**X**			X^b^		**X**	**X**	**X**

Notes:

a Ties within small subgroups in the intervention only (at weeks 3, 6, and 12 (i.e., 3 months, T2)). Otherwise, ties among all study participants were collected.

b Accelerometry data were not collected at T2.

## Data files and formats

Individual, deidentified participant data with data dictionaries, protocols, and annotated collection forms for the social network datasets and the larger GROW dataset to which they can be linked will be available to qualified investigators through BioLINCC: https://biolincc.nhlbi.nih.gov/studies/coptr/ starting August 2020. Enter ‘HLB02312020a’ in to the Search box.

Data files are in the comma-separated values (.csv) format. Sociometric data are stored as edgelists with the sender of each tie given in the first column, receivers and the associated tie characteristics denoted by subsequent column headers. Longitudinal data are structured in the ‘long’ format, where each row represents an ID timepoint (timepoints nested within ID number).

Study participants are identified by a six-digit numeric variable ‘NEWID’ in all datasets, and this ID is different from those used during the study. All potentially identifying information on participants within this study have been removed to safeguard participant confidentiality (names, locations, dates, etc.).

## Data details

**Table:** 

Response rate	The most conservative ‘intent-to-treat’ response rates, based on the full sample denominator, regardless of retention or administration issues or face-to-face intervention dose:
	*Full network dataset*
	Baseline: *n*=469/610 (76.9%) (delayed admin)
	Month 3: *n*=505 (82.8%)
	Month 12: *n*=522 (85.6%)
	Month 36: *n*=503 (82.5%)
	*Discussion and advice network dataset*
	Week 3: *n*=216/304 (71.1%)
	Week 6: *n*=213/304 (70.1%)
	Month 3: *n*=198/304 (65.1%)
	[Trial retention at 3 years was 550/610 (90.2%) based on primary outcome, child BMI]
Non-respondent bias	Differential SN completion rates:
	*Full network dataset*
	Baseline:
	Intervention *n*=251/304 (82.6%)
	Control *n*=218/306 (71.2%)
	Month 3:
	Intervention *n*=270/304 (88.8%)
	Control *n*=235/306 (76.8%)
	Month 12:
	Intervention *n*=263/304 (86.5%)
	Control *n*=259/306 (84.6%)
	Month 36:
	Intervention *n*=258/304 (84.9%)
	Control *n*=245/306 (80.1%)
	[Trial differential dropout based on primary outcome (child BMI) was 91.4% (*n*=278/304) for the intervention group; 88.9% (*n*=272/306) for the control group]
Theoretical grouping	These data were collected as part of a community-based pediatric obesity prevention intervention. At baseline, 304/610 parents were randomized to the intervention group and assigned to one of 30 small groups of approximately 10 participants. The intervention was delivered in these small groups of the same participants who met weekly for 12 weeks
Publications using this data	*Discussion and advice network dataset*
	Gesell et al. (2016)
	Gesell et al. (2020)
Data context	Randomized controlled trial
Respondents	Low-income parents with children 3 to 5 years of age, predominantly Hispanic
Longitudinal	Yes
	4 timepoints over 3 years 3 timepoints over 3 months
Temporality	In the Full Network Dataset the network is sparse, with a notable trend for participants in the intervention arm to form ties with other intervention participants (increased from 16.4% at baseline to 28.1 and 37.6% at 12 and 36 months, respectively), whereas participants in the control group formed ties at a notably slower rate and had comparatively fewer ties to participants in the intervention (increased from 10.1% at baseline to 12.0 and 17.1% at 12 and 36 months, respectively). (Paper under review)
	In the Discussion and advice network dataset, 34% of participants did not seek advice from anyone, 22% sought advice from one person, and 44% sought advice from two or more people. Seven participants listed the maximum of seven possible advice nominations
Analytical or pedagogical utility	Analysis of social network and health behavior data collected at the same time points Analysis of development of new social ties within the context of a group intervention, including comparison of intervention and control group, and comparison among 30 small intervention subgroups Analysis of multiplex ties
Known issues	There was a delay in the administration of the social network survey at baseline and network tie data were not collected from approximately 23% of the participants (intervention and control) at baseline. Standard multiple imputation techniques can be used to mitigate this issue, and the number of ties at baseline was very low for those with data Rolling recruitment and data collection over 1.5 years affected the utility of the Full Network dataset. It limited potential bidirectionality (e.g., earlier recruits could not nominate later recruits at baseline), and the temporal proximity of outcome collection between nominator and nominee is not always guaranteed
